# Dengue Fever in Burkina Faso, 2016

**DOI:** 10.3201/eid2401.170973

**Published:** 2018-01

**Authors:** Zékiba Tarnagda, Assana Cissé, Brice Wilfried Bicaba, Serge Diagbouga, Tani Sagna, Abdoul Kader Ilboudo, Dieudonné Tialla, Moussa Lingani, K. Appoline Sondo, Issaka Yougbaré, Issaka Yaméogo, Hyacinthe Euvrard Sow, Jean Sakandé, Lassana Sangaré, Rebecca Greco, David J. Muscatello

**Affiliations:** Institut de Recherche en Sciences de la Santé, Bobo-Dioulasso, Burkina Faso (Z. Tarnagda, A. Cissé, S. Diagbouga, T. Sagna, A.K. Ilboudo, D. Tialla, M. Lingani);; Ministère de la Santé Direction de la Lutte Contre la Maladie, Ouagadougou, Burkina Faso (B.W. Bicaba, I. Yaméogo, H.E. Sow);; Centre Hospitalier Universitaire Yalgado Ouédraogo, Ouagadougou (K.A. Sondo, J. Sakandé, L. Sangaré);; Sanofi Pasteur Limited, Toronto, Ontario, Canada (I. Yougbaré);; US Centers for Disease Control and Prevention, Ouagadougou (R. Greco);; University of New South Wales, Sydney, New South Wales, Australia (D.J. Muscatello)

**Keywords:** reemerging dengue fever, DENV-2, DENV-3, serologic diagnosis, molecular diagnosis, disease control, dengue, dengue fever, viruses, vector-borne infections, Burkina Faso, reemerging diseases

## Abstract

We report 1,327 probable cases of dengue in Burkina Faso in 2016. Of 35 serum samples tested by a trioplex test, 19 were confirmed dengue virus (DENV)‒positive: 11 DENV-2, 6 DENV-3, 2 nontypeable, and 1 DENV-2/DENV-3 co-infection. Molecular testing should be conducted to correctly identify causative agents in this complex infectious disease landscape.

Dengue is an emerging viral disease mainly found in the tropical and subtropical zones, and a major public health concern worldwide (*1*–*3*). Dengue fever is a mosquitoborne viral infection caused by 4 distinct dengue viruses (DENVs): DENV-1‒4. In some countries of sub-Saharan Africa, the circulation of all 4 viruses has been reported (*4*). However, availability of rapid tests and molecular diagnosis by reverse transcription PCR (RT-PCR) in resource-limited settings remains a challenge.

During October 29, 2016‒November 21, 2016, we screened 1,947 suspected dengue cases using a rapid diagnostic test (SD BIOLINE Dengue Duo, Standard Diagnostics, Seoul, South Korea), which detects DENV nonstructural protein 1 (NS1) and dengue-specific antibodies (IgM and IgG), in response to an outbreak of acute febrile illness in Burkina Faso. All patients with acute febrile illness during this period were suspected to have dengue; notably, some patients had biphasic fever with severe headache, myalgia, arthralgia, and rash. Patients who tested positive for NS1 or DENV antibodies were considered to have a probable DENV infection. All participants provided informed consent as specified by the Declaration of Helsinki, and approval of this study was obtained from the national ethics committee.

Of the 1,947 blood samples tested, 1,327 were positive for NS1, DENV antibodies, or both. Of the 13 country regions investigated, the central region, which includes the city of Ouagadougou, was the most affected, having 1,679 of the 1,947 suspected cases (case fatality ratio 1.2% [20/1,679]) and 1,307 of the 1,327 probable cases. Of the 20 deceased patients, 18 were positive for NS1 and 2 were positive for NS1 and DENV IgM. The outbreak peaked November 11‒14. Blood samples from 35 randomly selected patients were sent to the National Reference Laboratory for Influenza (Bobo-Dioulasso, Burkina Faso) for confirmation using the Centers for Disease Control and Prevention trioplex real-time RT-PCR protocol (*5*) followed by singleplex to identify the infecting DENV serotype. Of the 35 patient samples that were selected, 22 were positive for NS1, 3 were positive for both NS1 and IgG, 3 were positive for IgG, 2 were positive for both NS1 and IgM, 1 was positive for both IgM and IgG, and 4 were negative. Nineteen (54.3%) cases were positive for DENV, and no cases were positive for Zika or chikungunya viruses (Table). Eleven patients were infected with DENV-2, 6 were infected with DENV-3, and 1 patient was co-infected with DENV-2 and DENV-3. We submitted our samples to the World Health Organization Collaborating Centre for Arbovirus Reference and Research, Institut Pasteur de Dakar (Dakar, Senegal), which confirmed our results.

**Table:** Characteristics and rRT-PCR results of patients with dengue fever, Burkina Faso, 2016*

Variable	No. (%)	95% CI
Age, y		
2–9	2/35 (5.71)	NA
10–19	3/35 (8.57)	NA

20–29	8/35 (22.85)	NA
30–39	9/35 (25.71)	NA
40–49	9/35 (25.71)	NA
≥50	2/35 (5.71)	NA
Unknown	2/35 (5.71)	NA
Sex		
M	24/35 (68.57)	NA
F	8/35 (22.85)	NA
Unknown	3/35 (8.57)	NA
Molecular diagnostic		
rRT-PCR trioplex	19/35 (54.3)	34.78–70.78
rRT-PCR DENV-1–4	17/19 (89.4)	75.68–103.26
DENV-1	0 (0)	NA
DENV-2	11/19 (57.8)	35.69–80.09
DENV-3	6/19 (31.5)	11.57–51.57
DENV-4	0 (0)	NA
DENV-2 + DENV-3	1/19 (5.26)	4.74–15.26
Unknown serotype	2/19 (10.5)	7.2–23.52

*Of the 35 patient samples used, 31 were positive and 4 were negative by SD BIOLINE Dengue Duo (Standard Diagnostics, Seoul, South Korea). DENV, dengue virus; NA, not applicable; rRT-PCR, real-time reverse transcription PCR.

In Burkina Faso, dengue represents an added burden to an infectious disease landscape dominated by malaria; therefore, implementation of molecular diagnostic testing is urgently needed to identify the correct etiologic agent associated with the disease. The trioplex real-time RT-PCR detected 19 cases of DENV. A total of 3 serum samples positive for NS1 were negative by this assay. These negative results can be explained in part by declining viremia levels that became undetectable around the time of molecular testing, although testing with a larger representative sample size could have provided more information.

We found DENV-2 to be the dominant serotype in this outbreak, followed by DENV-3. No cases of DENV-1 or DENV-4 were found, although testing a larger number of specimens might have revealed the co-circulation of these DENV serotypes. Human cases of DENV-2 in Burkina Faso is supported by previous reports of DENV-2 circulating in mosquitoes (*6*). The presence of DENV-3 in Burkina Faso is not surprising, considering this serotype has been previously reported in the region; in 2009, DENV-3 was the main etiologic virus of the outbreak in Cape Verde, which affected >17,000 persons, and was reported in 6 persons in Senegal who traveled to Italy and died (*7*). DENV-3 was also detected in the DENV outbreak in Côte d’Ivoire in 2008 (*8*).

We speculate that increased international travel between neighboring countries and mosquito circulation has led to DENV-2 and DENV-3 successfully crossing the border into Burkina Faso. This pilot study shows DENV-2 and DENV-3 are both circulating in Burkina Faso and causing human disease. Molecular diagnostics, vector control strategies, and risk communication should be implemented in Burkina Faso in preparation for future outbreaks.
